# The State of the Tuberculosis Service

**Published:** 1914-03-28

**Authors:** 


### The Hospital

The Workers' Newspaper of Administrative Medicine and Institutional Life, Administration, National Insurance and Health.

No. 1448, Vol. LV. SATURDAY, MARCH 28, 1914. PRICE ONE PENNY.

### THE STATE OF THE TUBERCULOSIS SERVICE.

Would the general practitioner, if politely asked, and the whole matter fairly put before him, give up the "tuberculosis sixpence"? It may seem strange to discuss the case of a man consenting to curtail of his practice and emoluments, but nevertheless our belief is that in industrial districts, and in the North of England generally, the measure would come as a relief to the profession. For, as a general practitioner in a growing county borough frankly said to us recently, panel doctors have too much to do. "We are getting a name for being overworked and irritable," he added. The sad instance the other day of a man going about with advanced consumption, which was not detected for want of a physical examination, must have many parallels. In a large Northern centre eighty practitioners had left the panel before the middle of last year. A large "panel" contingent becomes so evident by its presence upon the doctor's premises that patients of higher social class are repelled. Of course, one must remember that the present rage among the working-class for sitting in a surgery of an evening may not last. Still, if a few Insurance patients were taken off private practitioners' shoulders, the latter might, seeing the present shortage of the medical profession, find it for some time to come a great relief. Now this could be secured by giving domiciliary treatment of the tuberculous to the tuberculosis officer, who in some districts, as is well known, has none too much to do. He would then have every tuberculous working-class subject under his eye, without omission. There would be no more disability of following up a dispensary patient who for a time needed treating at home. The practitioner would be relieved of the care of those cases of consumption too bad for the sanatorium or dispensary, and not quite bad enough for the hospital for advanced cases—a task, none too grateful, too, which the tuberculosis officer should presumably be able to do better than he.

We say presumably because a great many of these posts have been filled up not in the best possible way. The tendency now seems to be to appoint to the more highly-paid posts from those junior in this very junior service. This tendency is wrong; because whereas the administrative routine of tuberculosis officership can be picked up in a week, to become a reliable tuberculosis clinician is a matter of five years' exclusive experience. At present there are not a few misfits like the following. A fairly newly qualified gentleman—let us say, a provincial graduate—who happens to be of youthful and unimpressive personal appearance, and is therefore, as his parents realise, unlikely to hit the ignorant but important popular fancy as a practitioner, turns to public health work. He finds competition for posts as assistant medical officer of health very keen, and therefore becomes (be it noted that one of the dozen most intellectual men in the whole profession is a member of the service to be named) an "education officer." A colleague of his is a man over forty, married and with a family, who has lately left general practice. Now after a little there are mooted under the same authority assistant tuberculosis officerships. Getting earliest wind of these latter posts, the salary attaching to which is about £100 a year in excess of that they are receiving, these two gentlemen, obeying elementary instincts, approach among others their chief, the medical officer of health, with a view to getting them. They do get them; are sent for six weeks to a local smallpox hospital freshly converted into a sanatorium; turn over with wonder and secret dubiety the pictures of consumption of the larynx in their brand-new translated "Bandelier and Roepke"; read Dr. Camac Wilkinson's diatribes in the correspondence columns of the medical weeklies; and begin treating (on lines prudently laid down for them) dispensary patients, examining contacts, and the rest. After six months or a year, senior posts are advertised. One after the other, these gentlemen succeed again, often defeating those with a grounded knowledge of their subject. The class of work they will turn out is in no doubt. They have got into the bad habit of "hedging" and guessing over any but easy cases. This will continue, and the general practitioners of their neighbourhood will soon reckon them up pretty accurately. Just as nothing excites esoteric respect like clinical ability, so nothing like the lack of it, in a professor, arouses antagonism and ridicule.

But there will be more than unpleasantness caused to members of the tuberculosis service if many more weak-kneed clinicians are appointed. In one or two towns with provincial universities the proposal has been made to give the local medical faculty honorary clinical supervision over the municipal anti-tuberculosis work. The disadvantage of the resulting subordinate status to the tuberculosis officer is evident. For such as we have been describing a subordinate status would be very proper. But for others, say for those who have held posts at large consumptive hospitals and have worked at sanatoria, who can produce evidence of continued post-graduate study of throat diseases, we cannot see it as at all necessary. Surgical tuberculosis needing surgical treatment (which forms a mere fraction of the disease as a whole) must be left out of count: that must always go to the general or special hospital. But, as regards consumption, there are hardly any medical consultants in the provinces with experience requisite to make them real authorities. The subject and its home and foreign literature are far too vast to be kept up with by those also concerned with the whole wide field of internal medicine. What sort of a phthisiologist can he be who must also be cardiologist, neurologist, and one or two other things as well? Sir William Jenner in the 'seventies warned a certain consumptive celebrity against "trusting his vile body to the Davos doctors." Nowadays the boot is on the other leg. We all classify our cases as Turban has told us to, and listen to Sauerbruch at the International Medical Congress. The great want of the tuberculosis service at present is men of the stamp of Dr. Morland of Arosa. Attempts to officer it on the lines of orthodox officialism can only produce detriment all round.

